# In Memoriam: Professor Laurentiu M. Popescu (1944–2015)

**DOI:** 10.1186/s40169-015-0070-5

**Published:** 2015-09-10

**Authors:** Catalin G. Manole, Dragos Cretoiu

**Affiliations:** Department of Cellular and Molecular Biology and Histology, Carol Davila University of Medicine and Pharmacy, 8 Eroii Sanitari Blvd., 050474 Bucharest, Romania; Department of Molecular Medicine, Victor Babes National Institute of Pathology, Bucharest, Romania


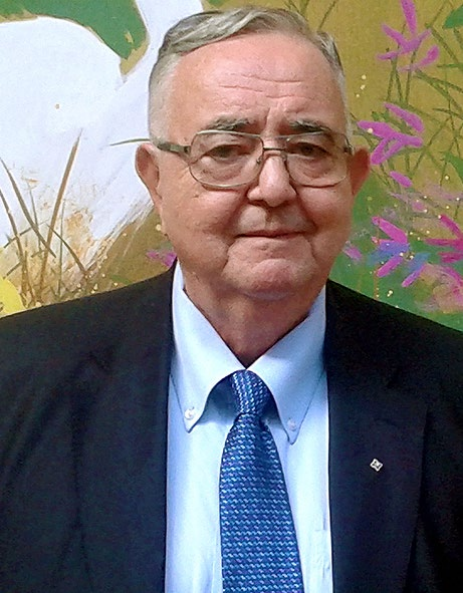
Laurentiu M. Popescu, a world renowned expert in the field of molecular medicine, Professor of Cellular and Molecular Medicine at Carol Davila University of Medicine and Pharmacy, Bucharest (Romania) and General Director of the National Institute of Pathology of Romania, passed away on August 3, 2015, at the age of 71.

Born on April 15, 1944, in Campulung, Romania, he devoted 48 years to research and teaching activities starting soon after graduation (as valedictorian) of ‘Carol Davila’ University of Medicine and Pharmacy. Backed by a constant progression in his academic career he became the Rector, between 1992 and 2004, of the ‘Carol Davila’ University (the oldest, largest and most prestigious in Romania), considered an example of progress and innovation in the 150 years of history of the University.

Professor Laurentiu Popescu was a very special and extraordinary scientist who gave a unique point of view to the international scientific community of medical and cellular sciences. He was awarded for his scientific achievements multiple prestigious prizes and was selected as member of many international scientific organizations. He was also the President of the Medical Science Section of the Romanian Academy of Sciences, President of the Romanian Academy of Medical Sciences, President of the Federation of the European Academies of Medicine, President of the International Society for Adaptive Medicine, and more.

In 2000 he founded the Journal of Cellular and Molecular Medicine. As Editor-in-Chief, he grew this journal very rapidly, transforming it, from a non-indexed journal into a world-class scientific journal (2014 Impact Factor 4.014).

While his academic career was pivoted around ‘Carol Davila’ University of Medicine and Pharmacy, a great part of the scientific achievements were centered on ‘Victor Babes’ National Institute of Pathology. Known as a visionary, he had a strong ability to inspire the people who worked with him. We will remember him for a myriad of virtues as wisdom, confidence, empathy and honesty, as well as outstanding motivation and communication skills. For us he has been a wonderful mentor, being extremely creative, and a really hard working person, endowed with great organizational skills. He was also a scientific advisor for numerous PhD and postdoctoral students sharing his outstanding experience and knowledge with all his disciples. Besides his impressive scientific knowledge he was also widely cultured, recognizing in one of his interviews “The idea to call the newly discovered cells telocytes came to me during a break at the Vienna State Opera”.

Professor Laurentiu M. Popescu’s vast scientific activity could be approximatively split in two main periods. The major scientific results of the first period were crystallized around the ideas of caveolae involvement in calcium regulation in smooth muscle, or around the relation between cGMP and vasodilation. The most fruitful and the most impressive one is the later part of Professor Laurentiu M. Popescu’s scientific activity dedicated to the discovery of a new type of interstitial cell in living organisms—Telocytes (first called “Popescu’s cells”)—and development of this top research field. This marvelous ‘scientific adventure’ started in 2005 as a typical case of serendipity when looking in pancreas interstitium for Interstitial Cells of Cajal, Professor Laurentiu M. Popescu attested and described the presence of a new type of interstitial cell, totally distinct from that one which intended to look for—Telocytes. Progressively, subsequent research conducted either by Professor Popescu’s group, or by other individual groups worldwide, documented the presence of telocytes within interstitium of many human and mammals cavitary and non-cavitary organs. He was an author of more than 300 scientific papers which have been published in international journals.

In 2009, his accomplishments were acknowledged with the highest honors by the government of Romania—The National Order “Star of Romania”.

The sudden disappearance of such a great personality creates a void in our hearts, and also strengthens the desire to continue the encouraging research developed together with him. As close co-workers, we will miss Professor Laurentiu M. Popescu’s great ideas and the capacity to assemble and organize a team and for bringing concepts to life. The keen productive working atmosphere surrounding him, was abundant of creativity and trust. He gave us values and rules and those were the pillars to construct that space of work and progress. The quintessence of his personality resides in the spirit of integrity, inherited by the team he exemplary lead. His tireless and motivational activity projected in perspective, thus permanently creating new aims for research.

Once the existence of Telocytes was attested, the saga of looking for their roles started, naturally. It appears that Telocytes are involved in tissue regeneration. If this will be confirmed, it could have a tremendous potential in cellular regeneration therapies. Perhaps this pursuit of telocytes roles will change the way we think physiology and/or pathology.

Instead of epilogue …

Francois Rabelais’ last words “I am heading to find-out the Biggest Perhaps” are best describing our Professor’s state of mind and his last majestic lesson for us.

